# Laparoscopic Resection of Appendiceal Schwannoma

**DOI:** 10.1155/2018/9191503

**Published:** 2018-08-15

**Authors:** Toru Imagami, Satoru Takayama, Yohei Maeda, Ryohei Matsui, Masaki Sakamoto, Hisanori Kani

**Affiliations:** Department of Surgery, Nagoya Tokushukai General Hospital, 2-52 Kouzouji-cho kita, Kasugai, Aichi 487-0016, Japan

## Abstract

**Background:**

Schwannoma arises from Schwann's cell of the neural sheath. Schwannoma of the large intestine, particularly of the appendix, is rare. We report a case of appendiceal schwannoma resected using laparoscopic surgery.

**Case Presentation:**

A 75-year-old man was referred to our hospital for abdominal fullness and nausea since 2 months. Abdominal CT revealed a well-demarcated oval mass of 25 mm at the tip of the appendix. Contrast-enhanced CT revealed a lesion with gradually enhanced contrast from the arterial phase to the equilibrium phase. Abdominal US revealed a well-demarcated hypoechoic tumor. Preoperative diagnosis indicated appendiceal mesenchymal or neuroendocrine tumor. Ileocecal resection with D3 lymph node dissection was performed. Pathological and immunohistochemical findings confirmed the diagnosis of appendiceal schwannoma.

**Conclusions:**

For determining the surgical procedure of nonepithelial tumor of the appendix, preoperative diagnosis of mesenchymal or neuroendocrine tumors is required. However, appendiceal schwannoma is extremely rare, and its characteristic findings have not yet been established. Accumulating cases of appendiceal schwannomas is necessary for improving imaging diagnosis and surgical treatment.

## 1. Introduction

Gastrointestinal mesenchymal tumors are classified into three types by immunohistochemical staining, namely gastrointestinal stromal tumor (GIST), leiomyoma, and schwannoma. Schwannoma arises from the Schwann's cell, which covers the peripheral nerves [1]. Gastrointestinal schwannoma is relatively rare and mostly occurs in the stomach, followed by that in the small intestine and in the colon or rectum [2], respectively. The frequency of occurrence of schwannoma in the stomach is 83%, whereas that in small intestine is 12% [3]. In recent years, submucosal tumors, including schwannoma, were incidentally detected in a screening colonoscopy. Although there are some reports available on the endoscopic diagnosis, endoscopic resection, and surgical resection for colorectal schwannoma, those on appendiceal schwannoma are extremely limited; thus, clinical features of schwannoma have not yet been clarified. To the best of our knowledge, only a few cases of appendiceal schwannoma have been reported on PubMed [4–9]. In this report, we describe our case of appendiceal schwannoma resected using laparoscopic surgery, supported with a literature review.

## 2. Case Presentation

**Figure 1:**
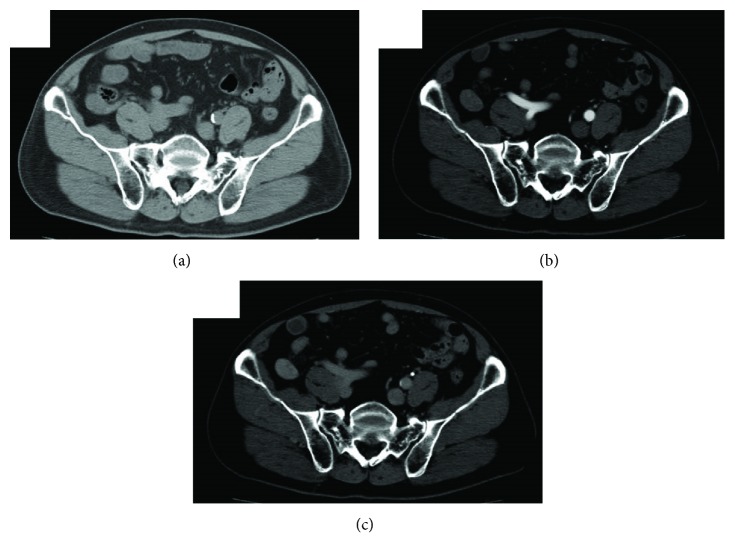
Abdominal CT showed well-defined isodensity oval mass at the tip of appendix (a). In contrast-enhanced CT, the lesion was gradually enhanced homogeneously from arterial phase (b) to equilibrium phase (c).

**Figure 2:**
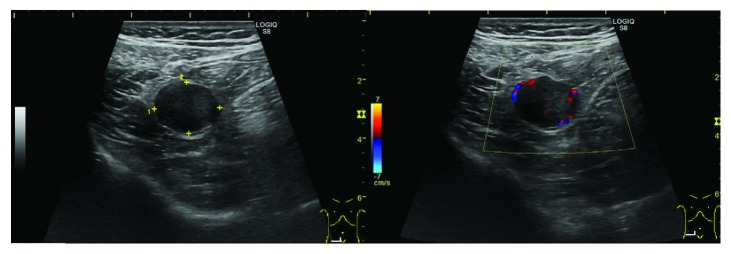
Abdominal ultrasonography showed that a well-demarcated hypoechoic mass of 22 mm × 18 mm × 18 mm. There were cystic area and blood flow sign.

A 75-year-old man was referred to our hospital for abdominal fullness and nausea since 2 months. He had a medical history of hypertension and hyperlipidemia and a surgical history of the right inguinal hernia. The patient's laboratory findings were within normal limits. Abdominal computed tomography (CT) revealed a well-demarcated oval isodensity mass of 25 mm at the tip of his appendix. Contrast-enhanced CT revealed a lesion with gradual homogeneous contrast enhancement from the arterial phase to the equilibrium phase (Figure 1). No abnormal findings were found in the root to the middle of the appendix. Abdominal ultrasonography (US) revealed a well-demarcated hypoechoic tumor. The tumor size was 22 mm × 18 mm × 18 mm, with some cystic area and blood flow (Figure 2). Colonoscopy findings were normal. The patient's symptoms naturally alleviated during examination period.

Preoperative diagnosis indicated appendiceal neuroendocrine tumor (NET) G1 or gastrointestinal mesenchymal tumors, such as GIST. Malignancy could not be ruled out; therefore, laparoscopic ileocecal resection with D3 lymph node dissection was recommended. Intraoperative findings revealed a well-demarcated tumor at the tip of the appendix, with no invasion into the surrounding tissue. This observation was similar to the preoperative imaging findings. According to another intraoperative finding, dissecting the adhesion between the terminal ileum and the peritoneum, which was the effect of the past herniorrhaphy, was necessary. The operation time was 167 min, and the amount of blood loss was 100 ml.

**Figure 3:**
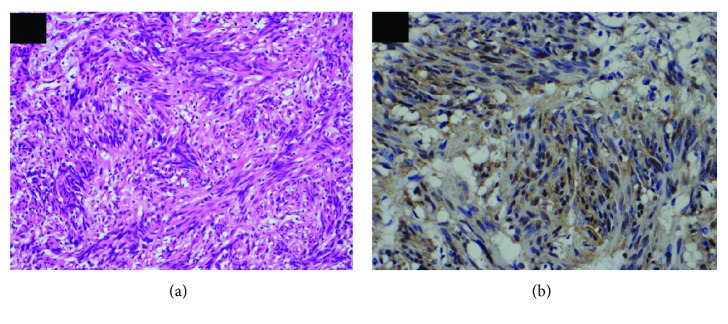
Pathological finding in HE staining showed spindle-shaped heterotypic cells proliferating in a bundle (a). In immunohistochemical studies, the nucleus stained by S-100 protein (b).

Pathological findings revealed a well-demarcated tumor originating from the muscular layer at the tip of the appendix and spindle-shaped heterotypic cells proliferating in a bundle. Vascular invasion and lymph duct invasion were not detected. No tumor cells were found in the dissected lymph node. Immunohistochemical studies revealed negative values for KIT and CD34 and positive values for S-100 protein (Figure 3), which confirmed the schwannoma of the appendix. The patient was discharged on the 9th day after surgery without any complication requiring medical treatment. The patient is presently doing well without any evidence of recurrence at 3 months after surgery.

## 3. Discussion

Verocay [3] first described gastrointestinal schwannoma in 1910 [1, 10], with two histological growth patterns, namely, “Antoni A” and “Antoni B” [3]. The Antoni A pattern is characterized by long spindle cells that create a palisading pattern, and when well developed, this palisading feature can create the so-called “Verocay bodies” [2]. The Antoni B pattern is characterized by a loose arrangement of cells, with varying degrees of myxoid and hyaline degeneration [2]. On imaging, the more vascular Antoni A areas appear as enhancing solid components, whereas Antoni B areas frequently occur as nonenhancing cystic or multiseptate components [2]. According to the report of Hirota et al. [11] in 1998, gastrointestinal mesenchymal tumors are now classified as GIST, leiomyoma, and schwannoma by immunohistochemical staining.

Schwannoma can occur at any location throughout the body along the peripheral nerves [1, 12]. Gastrointestinal schwannoma most commonly originates in the Auerbach's nerve plexus and, at times, in the Meissner's nerve plexus [3, 13]. Schwannoma of the large intestine is rare; however, the cases of the accidental discovery of schwannomas of the colon and rectum have increased with the development of endoscopic examination techniques in recent years. Nonetheless, appendiceal schwannoma is extremely rare, with only a few cases reported on PubMed [4–9].

Gastrointestinal schwannoma assumes the form of a submucosal tumor. The incidence of schwannoma has been reported to be 2%–6% of all submucosal tumors of the intestine [12, 14]. In the primary gastrointestinal submucosal tumor, GIST and NET G1 may be malignant; therefore, preoperative diagnosis of gastrointestinal submucosal tumors is considered important although this is challenging as these tumors are often confused with other neoplasms despite advances in the imaging studies [2]. Inagawa et al. have reported that the rate of accurate preoperative diagnosis for schwannoma is only 15.2% [1]. Recently, the usefulness of endoscopic US (EUS) and endoscopic US-guided fine-needle aspiration (EUS-FNA) has been reported for the diagnosis of gastrointestinal submucosal tumors [12, 15, 16], although it is often difficult for the appendiceal lesions.

Regarding appendiceal NET G1, some cases of appendicular goblet cell carcinoid, which exhibit pathological characteristics of both carcinoid and adenocarcinoma, have been recently reported. Goblet cell carcinoma of the appendix requires a treatment approach similar to that required for a colorectal malignant tumor. Some reports of additional resection surgery for lymph node dissection after appendectomy for appendiceal goblet cell carcinoma are available [17]. Marudanayagam et al. [18] have reported that carcinoid was observed in 0.52% of specimens of appendectomy. The detection of these neoplasms at preoperative imaging was important because it may change the surgical approach and obviate additional surgery.

Gastrointestinal schwannoma and GIST show similar imaging findings [2]; however, several different characteristic findings of gastrointestinal mesenchymal tumors and NET have been clarified. The typical CT finding of gastrointestinal schwannoma is well-defined, round-to-oval, homogeneous, and low-attenuation tumor [2, 3, 19]. More homogeneous attenuation, more often exophytic growth pattern, and the presence of perilesional lymph nodes are significantly different in gastrointestinal schwannoma than in GIST [2, 20]. Contrast-enhanced CT revealed delayed enhancement pattern, and the contrast effect in the equilibrium phase of schwannoma was significantly enhanced compared with those of GIST and leiomyoma [19]. Small GIST typically grows into a well-demarcated exophytic mass of homogeneous density, and large GISTs of ≥5 cm are often heterogeneous with necrosis, hemorrhage, and cystic degeneration as they grow larger [19]. Gastrointestinal carcinoid is rarely detected on CT, and pancreatic NET exhibit isodensity [21]. Diffuse mucosal hypertrophy is characterized by appendiceal NET [22]. The characteristic of NET is the contrast pattern from the arterial phase to the equilibrium phase [21]. Typical EUS findings of the gastrointestinal schwannoma include well-demarcated hypoechoic lesions in the 4th tumor layer, which is similar to that of GIST and leiomyoma [16]. In contrast, NET is a hypoechoic lesion in the 2nd or 3rd tumor layer. Evaluation of the localized layer of tumor in US is considered to be effective for distinguishing between the mesenchymal tumor and NET. CT findings of mesenchymal tumor and NET are shown in Table 1, and US findings are shown in Table 2.

[^18^F]Fluorodeoxyglucose (FDG) positron emission tomography (PET) has been used for detecting malignant tumor. However, there are several reports of gastrointestinal schwannoma with increased FDG uptake, among which is a case of appendiceal schwannoma detected by FDG-PET reported by Nishio et al. [7, 23, 24]. In this regard, FDG-PET cannot help confirm the preoperative diagnosis of appendiceal schwannoma.

In our case, abdominal CT revealed a well-defined oval mass of 25 mm at the tip of the appendix. Contrast-enhanced CT revealed the mass with gradual homogeneous contrast enhancement from the arterial phase to the equilibrium phase. Several 5 mm lymph nodes surrounding the ileocolic artery were identified. Abdominal US revealed a well-demarcated hypoechoic hypervascular mass and some cystic area. The CT findings were similar to the known findings of schwannoma. In the US findings, the partial cyst region was different from that reported in the general findings of schwannoma, suggesting the possibility of it depicting the Antoni B area. In the abdominal US, the wall structure was not maintained, and the evaluation of the localized layer of the wall was difficult. Abdominal US may be useful for the diagnosis rather than EUS if the digestive tract wall structure of the appendix can be depicted. According to Bucher et al. [25], colonoscopy for patients with accidentally discovered appendiceal tumors is recommended because of the higher rate of synchronous colon cancer. In our case, colonoscopy findings were normal.

The standard treatment for schwannoma is complete resection, and several cases of laparoscopic surgery for schwannoma of the large intestine have been reported [3, 4, 12, 15]. When the tumor size is ≥5 cm, it is not resected because of the possibility of malignantization [3, 13]. The results of both chemotherapy and radiotherapy remain uncertain [4, 15]. Lymph node dissection is not recommended for gastrointestinal schwannoma [15, 16] and GIST. The guideline of the Japan Society of Clinical Oncology (JSCO) recommend surgical procedure for appendiceal NET depending on the size of the tumor, presence of invasion findings, and the presence of suspected metastatic lymph node.

In this case, our preoperative diagnosis was appendiceal NET or mesenchymal tumor with a >2 cm mass. According to the JSCO guideline, ileocecal resection or right hemicolectomy with lymph node dissection was recommended. The presence of enlarged lymph nodes also contributed to this recommendation of lymph node dissection. Currently, regarding the diagnosis of the nonepithelial tumor of the appendix, such as mesenchymal tumor and NET, it is necessary to decide a surgical treatment according to both gastrointestinal mesenchymal tumor and NET guidelines.

## 4. Conclusion

We describe a case of appendiceal schwannoma resected using laparoscopic surgery. There are few reports of gastrointestinal mesenchymal tumors, including appendiceal schwannoma; therefore, characteristic clinical findings of appendiceal schwannoma remain unclear. Thus, accumulating cases of appendiceal schwannoma are warranted for improving the imaging diagnosis and surgical treatment of appendiceal schwannoma.

## Figures and Tables

**Table 1:** Characteristic CT findings of mesenchymal tumor and NET G1.

	Density	Shape	Margins	Enhancement pattern	Enhancement phase
Schwannoma	Isodensity	Round or oval	Well-defined	Homogeneous	Delayed phase
GIST	Isodensity	Round or oval irregular	Well-defined	Homo or heterogeneous	Nonspecific
Leiomyoma	Isodensity	Round or oval	Well-defined	Homogeneous	Nonspecific
NET G1	Bowel wall: isodensity	Appendix: diffuse mural thickening	Well-defined	Homogeneous	Arterial phase

**Table 2:** Characteristic US or EUS findings of mesenchymal tumor and NET G1.

	Layer origin	Margins	Feature with US	
Schwannoma	4th layer	Well-defined	Hypoechoic	Homogeneous
GIST	4th layer	Well-defined	Hypoechoic	Homo-heterogeneous
Leiomyoma	4th layer	Well-defined	Hypoechoic	Homogeneous
NET G1	2nd~3rd layer	Well-defined	Hypoechoic	Homogeneous
